# The importance of bioceramics and computed tomography in the late clinical management of a horizontal root fracture: A case report

**DOI:** 10.4317/jced.56585

**Published:** 2020-05-01

**Authors:** Caroline-Felipe-Magalhães Girelli, Carolina-Oliveira de Lima, Mariane-Floriano-Lopes-Santos Lacerda, Renato-Girelli Coellho, Frank-Ferreira Silveira, Eduardo Nunes

**Affiliations:** 1 MSc. Department of Dentistry, Pontificial Catholic University of Minas Gerais, Brazil; 2 MSc. Department of Dentistry, State University of Rio de Janeiro,UERJ- RJ; 3 DDs. Department of Dentistry, Juiz de Fora Federal University, UFJF/GV- MG; 4 DDs. Department of Dentistry, Pontificial Catholic University of Minas Gerais, Brazil

## Abstract

Root fractures resulting from dental trauma involve dentin, cementum and pulp. The present study aimed to demonstrate the importance of cone-beam computed tomography (CBTC) and bioceramics in the correct planning and intervention of a horizontal root fracture case in tooth 11 with late treatment in an 18-year-old patient. Clinical and radiographic examinations revealed tooth displacement, pain on vertical percussion and images suggestive of a horizontal root fracture. Pulp necrosis was diagnosed and CBTC was requested for treatment planning. Subsequently, endodontic treatment was performed using a bioceramic apical plug. A 2-year follow-up indicated the absence of root resorption and normal periodontal and periapical tissues. It was concluded that endodontic treatment associated with the use of bioceramics and the aid of CBTC is an effective therapeutic option in cases of permanent horizontal root fractures.

** Key words:**Bioceramics, Cone-beam computed tomography, dental Injuries, root fracture.

## Introduction

Horizontal or oblique root fractures, also termed intra-alveolar fractures, are characterized by the rupture of rigid root structures, divided into two segments, apical and coronary. These fractures represent 0.5-7% of all dental injuries and are mainly observed in the anterior maxilla, affecting the incisors, and more often, teeth presenting complete rhizogenesis (1).

According to International Association of Dental Traumatology recommendations, initial horizontal fracture treatment consists in repositioning the coronary fragment, flexible splinting for four weeks and clinical and radiographic follow-up (2). However, the frequency of pulp necrosis of the coronary fragment of the traumatized tooth ranged from 22 to 26% (1), requiring endodontic treatment up to the fracture line level (3).

 In this context, the diagnosis and evaluation of horizontal fractures through the use of 3D images are important in determining treatment complexity. Cone-beam computed tomography (CBTC) has been widely used in endodontics, as it provides images in the axial, coronal and sagittal directions, allowing for dental structure visualization without overlapping images and the detection of the exact fracture location and dental fragment displacement (4).

Bioceramics have been extensively applied in root fracture treatments, as they display adequate sealing ability and biocompatibility and induce mineralized barrier formation in periradicular tissues by hydroxyapatite formation, enabling the creation of an apical plug with reduced treatment time. These compounds are also easy to apply and do not alater the color of the dental crown (5).

Given the above, the aim of this study was to describe a clinical horizontal root fracture case treated by applying bioceramics and CBTC, with a 2-year follow-up demonstrating success.

## Case Report

**Figure 1:**
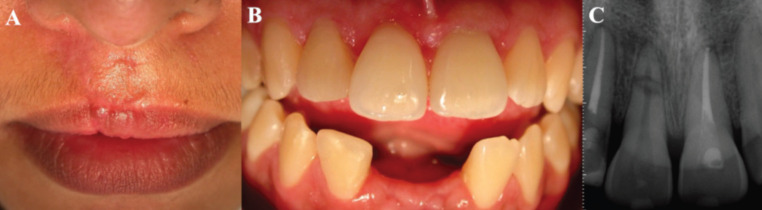
A) Scar on the right upper lip due to trauma. B) Absence of teeth 31 and 41, avulsed. C) Initial x-ray suggestive of apical horizontal fracture.

A 18-year old Caucasian female patient attended the dental office complaining of discomfort in tooth 11. During the anamnesis, the patient reported having had a bicycle fall within three months. A clinical examination revealed a scar on the right upper lip (Fig. 1A) and the absence of teeth 31 and 41, avulsed as a result of the fall (Fig. 1B).

Tooth 11 was found to be out of the occlusal plane (Fig. 1B), with grade 2 mobility and marked painful response to vertical percussion. Pulp sensitivity tests with thermal stimulation (cold) using Endo-Ice® (Maquira Dental, Maringá, Paraná, Brazil) and an electrical test with Pulp-Tester® device (Odous De Deus, Belo Horizonte, Minas Gerais, Brazil) were negative. A periapical radiographic examination revealed a radiolucent imaging in the apical segment of the root, compatible with a root fracture (Fig. 1C).

In order to support correct treatment planning, a CBTC was requested. CT scans were performed (I-Cat®, Imaging Sciences International, Hatfield, Pensilvânia, EUA), operating at 120KV, 36.12mAs, exposure time of 40 s, 0,2mm voxel size and Field of view (FOV) of 6x6 cm.

**Figure 2:**
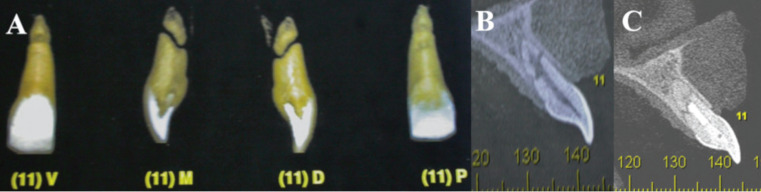
A) Tomographic image of tooth 11, coronal and sagittal sections. B) Sagittal section demonstrating fracture extension, with fragment separation. C) 2-year proservation with fracture repair by mineralized tissue interposition, suggestive of bone deposition healing.

The images were analyzed, and coronal and sagittal sections confirmed the separation of several fragments along the entire transverse root extension, as well as the absence of root resorption resulting from the fracture (Fig. 2A,B).

**Figure 3:**
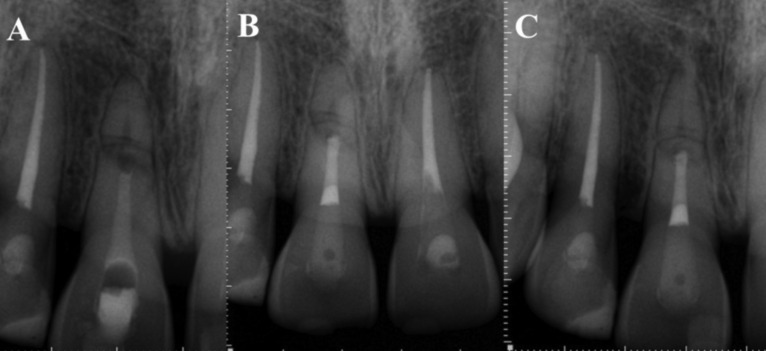
A) Intracanal medication, B) Bioceramic obturation and 2 mm cervical obturation with thermoplastic gutta percha - 1 year control C) Proservation after 2 years.

After the patient’s free and informed consent, a coronary access was performed under absolute isolation by means of dental floss ties and a Top Dam® gingival barrier seal (FGM, Joinville, SC). The working length was precisely determined by the CBTC, at 17 mm, with the palatal face of the tooth as the apical end the (Fig. 2A). Mechanized instrumentation was performed using the Protaper Next system up to the X5 file (Dentsply Tulsa Dental Specialties, Tulsa, OK, USA). Copious irrigation with 5.25% sodium hypochlorite was performed and during each instrument exchange using a 5 mL Luer syringe equipped with NaviTip 30 gauge needles (Ultradent Products Inc., Indaiatuba, SP), inserted into the canal up to 2 mm from the WL. After complete instrumentation, the canal was irrigated with 5 mL of ethylenediaminetetraacetic acid (EDTA, Formula and Action) at 17% followed by application of an intracanal calcium hydroxide medication (Ultracal, Ultradent, South Jordan, UT) for 10 days (Fig. 3A). After the placement of a delay dressing, a slight wear was made on the incisal edge of the tooth.

When no signs and symptoms were observed, a 4 mm apical plug was created using Bio C repair Bioceramic cement (Angelus, Londrina, Paraná), with the aid of condensers (Odous De Deus, Belo Horizonte, Minas Gerais, Brazil). Subsequently, the two coronary millimeters were filled with thermoplastic gutta percha, inserted with an Obtura pistol (Sybron Endo, Orange, CA, USA) (Fig. 3B).

Two years later, a clinical and radiographic examination was performed and no signs or symptoms of therapy failure were observed (Fig. 3C). A new tomographic examination was requested to follow up on the apical fragment without endodontic treatment. Mineralized tissue interposition was observed in the fracture region, without any resorptive alterations. Periapical region integrity was maintained, with no periradicular lesion or ligament thickening (Fig. 2C).

## Discussion

The management of horizontal root fractures is influenced by several factors, such as root formation stage, coronary fragment dislocation degree, interval between trauma and treatment and fracture location (6). When fractures are located in the apical third, the incidence of pulp vitality is of 96%, in the middle third, 86% and in the cervical third, 20% (7). Once necrotic, IADT guidelines recommend endodontic treatment (8,9).

However, clinical and radiographic follow-up of the traumatized tooth is required, as the negative response to pulp tests may be temporary, with fragment revascularization (10). This is due to the fact that the fractured area can act as an escape path for possible collateral circulation, thus aiding in maintaining pulp vitality for a certain period of time (11). After 3 months of proservation, if the tests are still negative and/or a radiographically radiolucent image appears between the fracture lines, endodontic treatment of the coronary third only is indicated (6,8).

In line with the literature, the present study reports endodontic coronary fragment treatment was performed after negative pulp test responses 3 months after the trauma and also due to the presence of vertical percussion pain. It was decided to maintain the apical fragment, without, however, performing any manipulation, due to its revascularization ability (6,8).

The use of a calcium hydroxide paste has been recommended as an intracanal medication in the endodontic treatment of fractured teeth, in order to promote a barrier at the apical region of the coronary fragment to accommodate the obturator material. However, such medication requires prolonged treatment and several medication changes (3,12). 

New bioceramics, based on calcium silicate, have been used as an alternative to calcium hydroxide, as they are extremely biocompatible (non-toxic) and also establish a chemical bond with dentin during the hardening process, forming hydroxyapatite. The material interacts with stem cells from periapical tissues, producing a biological seal and inducing the repair process (4).

 Materials such as MTA (mineral trioxide aggregate) display biological and chemical properties that accredit it for use as an apical buffer. Disadvantages such as the possibility of teeth discolouration, relatively difficult handling, long hardening time and the release of heavy metals, have been used to question their use (4).

Recently introduced in the market, Bio-C REPAIR (Angelus, Londrina Paraná, Brazil), in addition to displaying the benefits of bioceramic formulation, such as tissue regeneration induction, bactericidal action and bacterial infiltration inhibition, also presents a significant advantage over traditional cements that do not require manipulation. Its ready-to-use presentation facilitates product removal for on-site preparation, simplifying this procedure while also saving time (13)

As the case reported herein is a root fracture, which requires a repair process conducted on the separation line, the new bioceramic material was used. After two years of proservation, the patient presented no painful symptoms, no crown discoloration and radiographically, no periapical lesion, which characterizes a successful treatment so far.

Still regarding the proservation point of view, one aspect that should be observed concerning the fracture line is the presence of external root resorption secondary to root fractures, which may be present in up to 60% of cases. Radiographically, this defect may take up to two weeks to be visualized and still be inaccurate (10).

The two-dimensional nature of periapical radiographs limits the information that can be obtained. Image quality depends on factors such as radiographic cylinder angulation, overlapping anatomical structures and other patient-related factors, raising doubts regarding the association of resorption with the root surface and its internal or external origin (14). 

Currently, CBTC has been applied in the diagnosis of or during endodontic treatment of selected cases, where after the use of periapical radiography and its variables, clinician doubts are not answered (15). Several authors (14,15) point out that the main advantage of CBTC over conventional radiography is its three-dimensional image, which allows greater precision and may clarify the nature of a resorptive defect. Thus, it allows for rectified diagnoses, leading to the use of appropriate techniques for a favorable prognosis. In addition, CBTC enables easily acquired highly accurate imaging, and a low dose of effective radiation when compared to medical tomography. Corroborating this statement, Orhan *et al.* defined that 3D visualization has been shown to be an excellent resource for resorption diagnosis, with superior results when compared to conventional periapical radiographs.

Different sizes of voxel and FOV may influence the image quality of a computed tomographic scan and the radiation dose delivered to the patient. Small voxel and reduced FOV used in CBCT scans are known to generate images with higher resolution compared with another parameters; this is an important attribute for detecting details, such as root fracture. Nevertheless, a decreased of voxel size, exposes the patient to greater amounts of radiation. Thus, the effort to get better quality images represents a major biological cost (16). However, this limitation can be overcome by the use of reduced FOV which reduces the absorption of radiation by the patient (17).

In order to verify the occurrence of this defect in the fracture line of the present report, the CBCT was requested two years after endodontic treatment completion. Considering the ALADA (as low as diagnostically accepTable) principle and in line with the literature, they were used as exposure parameters required in the exam- reduced FOV (6x6 cm) and small voxel size (0.2 mm). No external root resorption was identified. However, a healing process was noted through the interposition of a mineralized tissue compatible with bone tissue.

Another noteworthy aspect is the type of healing that occurs in the fracture line, which can be by interposition of bone tissue, when fragments are significantly separated, by interposition of connective tissue, through the formation of cementum and dentin callus, association of bone and connective tissue, and interposition of granulation tissue (when the pulp goes into necrosis). Hard tissue healing is the best result to be expected, while the presence of granulation tissue represents an unfavorable inflammatory state (10).

Given these results, root canal treatment associated with the use of bioceramics may be an effective therapeutic option in cases of horizontal root fractures of permanent teeth. In addition, CBTC examination should be considered a viable option for the diagnosis, location and definition of root fracture extension to assist in endodontic therapy.
